# CRISPR Gene-Editing Combat: Targeting AIDS for total eradication

**DOI:** 10.12669/pjms.40.10.10257

**Published:** 2024-11

**Authors:** Maryam Nasrumminallah, Mohammad Hadif, Kashaf Fatima

**Affiliations:** 1 Maryam Nasrumminallah, House Officer, Dow University Hospital, Karachi, Pakistan; 2 Mohammad Hadif, Dow International Medical College, Karachi, Pakistan; 3 Kashaf Fatima, Dow International Medical College, Karachi, Pakistan

HIV/AIDS, a sexually transmitted disease, targets the immune system, weakening defences against infections and cancers. The most advanced stage, acquired immune deficiency syndrome (AIDS), can take years to develop but demonstrates severe long-term clinical manifestations.^1^ Approximately 38 million people are infected with HIV and 800,000 people die from HIV-related conditions every year making it a major global health burden. There are currently no effective vaccines, nor is there a cure, for HIV infections combined Anti Retroviral Therapy(cART), the current golden standard treatment for HIV infections, is effective in controlling viral replication.^2^ However, cART cannot efficiently eliminate latent viral reservoirs, making HIV-1/AIDS a chronic and incurable disease. Moreover, the high costs of therapy, the necessity to take the drug for the rest of the life, its side effects such as cardiovascular, cerebrovascular and neurological complications as well as chronic liver disease and drug resistance should also be considered in HIV-1/AIDS treatment. Therefore, new therapeutic strategies to inhibit HIV-1 replication and eliminate latent reservoirs are required urgently.^3^,^4^

Lately, scientists have been working to gain a deeper understanding and conducting thorough research into potential treatments for AIDS using clustered regularly interspaced short palindromic repeat (CRISPR)/CRISPR-associated nuclease 9 (Cas9) system, gene editing technology.^3^ In 2013, CRISPR/Cas9-based strategy for HIV/AIDS treatment was first tested by Ebina et al. which successfully inhibited HIV-1 gene expression in Jurkat cells.^3^,^5^ The “BERLIN PATIENT” with AIDS and Acute Myelocytic Leukemia (AML), who was functionally cured following a bone marrow transplant including a CCR5 ∆ 32 genotype, demonstrates the encouraging outcomes of recently developed gene therapy and further suggests that AIDS may be eradicated shortly. Zhang et al. discussed similar proof of curative therapy which was presented more recently in a clinical report by Gupta et al. on the “London patient” with Hodgkin’s lymphoma and AIDS.^4^ Consequently, Gene therapy is currently employed in the treatment of diverse genetic conditions, including beta thalassemia, hemophilia, and other monogenic diseases.^4^ The researchers at Amsterdam University are diligently exploring Nobel prize-winning CRISPR gene editing technology to achieve complete treatment for AIDS, building on the encouraging progress they’ve witnessed. They describe its mechanism as similar to scissors, where CRISPR technology cuts DNA at a microscopic level, enabling the elimination of troublesome segments. Moreover, Excision BioTherapeutics’ findings suggest promising potential for their technology, with three HIV volunteers showing no significant side effects after 48 weeks of treatment.^6^

Gene editing technology indeed holds tremendous promise for addressing diseases like AIDS. While there’s no complete cure for AIDS yet, advancements in gene editing, such as CRISPR-Cas9, offer hope for potentially eradicating the disease in the future through targeted modifications to the genetic material of affected individuals. Continued research and development in this field, employing extensive controlled trials and leveraging testing technology across diverse populations and conditions, could pave the way for significant breakthroughs in the battle against AIDS. The hope is that the technology will eventually produce results that are more powerful than those that have been honed over billions of years of evolution. Hence, advancing the development of CRISPR gene editing actively requires the engagement of medical institutions, experts, academic institutions, companies, and third-party operators to propel its progress further.
